# Veno-arterial extracorporeal membrane oxygenation and targeted temperature management in tricyclic antidepressant-induced cardiac arrest: A case report and literature review

**DOI:** 10.1097/MD.0000000000024980

**Published:** 2021-03-05

**Authors:** Kaoru Ikejiri, Yuichi Akama, Yohei Ieki, Eiji Kawamoto, Kei Suzuki, Kazuto Yokoyama, Ken Ishikura, Hiroshi Imai

**Affiliations:** Emergency and Critical Care Center, Mie University Hospital, 2-174 Edobashi, Tsu city, Mie 514-8507, Japan.

**Keywords:** tricyclic antidepressant, targeted temperature management, veno-arterial extracorporeal membrane oxygenation

## Abstract

**Rationale:**

Cardiotoxicity is a common cause of death in tricyclic antidepressant (TCA) intoxication. Veno-arterial extracorporeal membrane oxygenation (VA-ECMO) is effective in critically ill poisoned patients who do not respond to conventional therapies, and targeted temperature management (TTM) is associated with improved neurological outcomes and mortality in comatose out-of-hospital cardiac arrest survivors. However, few reports have documented cases of TCA intoxication that required intensive care, including VA-ECMO or TTM.

**Patient concerns:**

A 19-year-old Japanese man with a history of depression was brought to our hospital because he was in a comatose state with a generalized seizure. Before admission, he had taken an unknown amount of amitriptyline.

**Diagnosis:**

After intubation, the electrocardiogram (ECG) displayed a wide QRS complex tachycardia, and the patient suffered from cardiovascular instability despite intravenous bolus of sodium bicarbonate. At 200 minutes after ingestion, he experienced a TCA-induced cardiac arrest.

**Interventions:**

We initiated VA-ECMO 240 minutes after ingestion. The hemodynamic status stabilized, and the ECG abnormality improved gradually. In addition, we initiated targeted temperature management (TTM) with a target temperature of 34°C.

**Outcomes:**

Twenty seven hours after starting the pump, the patient was weaned off the VA-ECMO. After completing the TTM, his mental status improved, and he was extubated on day 5. He was discharged on day 15 without neurological impairment, and the post-discharge course was uneventful.

**Lessons:**

First, VA-ECMO is effective in patients with TCA-induced cardiac arrest. Second, routine ECG screening during VA-ECMO support is useful for assessing the timing to wean off the VA-ECMO, as well as the degree of cardiotoxicity. Third, TTM is safe in comatose survivors of cardiac arrest caused by severe TCA intoxication.

## 1 Introduction

Tricyclic antidepressants (TCAs), including amitriptyline, are commonly used in the management of depression. However, overprescription or inappropriate use of TCAs can lead to coma and seizure. Large TCA overdoses are cardiotoxic, resulting in fatal arrhythmia and refractory hypotension, which is one of the most common causes of death in TCA intoxication.^[1]^

Veno-arterial extracorporeal membrane oxygenation (VA-ECMO) is a life-supporting procedure that can be considered for critically ill patients with refractory cardiogenic shock, pulmonary embolism, hypothermia, and cardiac arrest.^[2]^ Specifically, VA-ECMO is effective in critically ill poisoned patients who do not respond to conventional therapies.^[3]^ Targeted temperature management (TTM) is associated with improved neurological outcomes and mortality in comatose out-of-hospital cardiac arrest survivors.^[4]^ However, only a few reports have documented cases of TCA intoxication that require intensive care, including VA-ECMO or TTM. Here, we report a case of amitriptyline intoxication. The patient, who had suffered cardiac arrest caused by the drug's cardiotoxicity, was successfully resuscitated using both VA-ECMO and TTM.

## 2 Case presentation

A 19-year-old Japanese man with a history of depression was brought to our hospital in a comatose state with a generalized seizure. His family had received a phone call from him, informing them that he had taken an unknown amount of amitriptyline. His family found the patient in an altered mental status with a seizure and called for an emergency medical service. On admission (150 minutes after ingestion), his initial body temperature, pulse rate, blood pressure, and SpO_2_ were 36.1°C, 86 bpm, 92/39 mm Hg, and 98%, respectively. He also presented a comatose status (Glasgow Coma Scale score 3; E1V1M1), along with generalized tonic-clonic seizure. Laboratory analysis detected a substantially elevated serum lactate level (>20 mmol/L). His urine test result was positive for TCA. The admission serum concentrations of amitriptyline and its active metabolite nortriptyline, 2902 ng/ml and 712 ng/ml, respectively, were measured at a later date.

**Figure 1:**
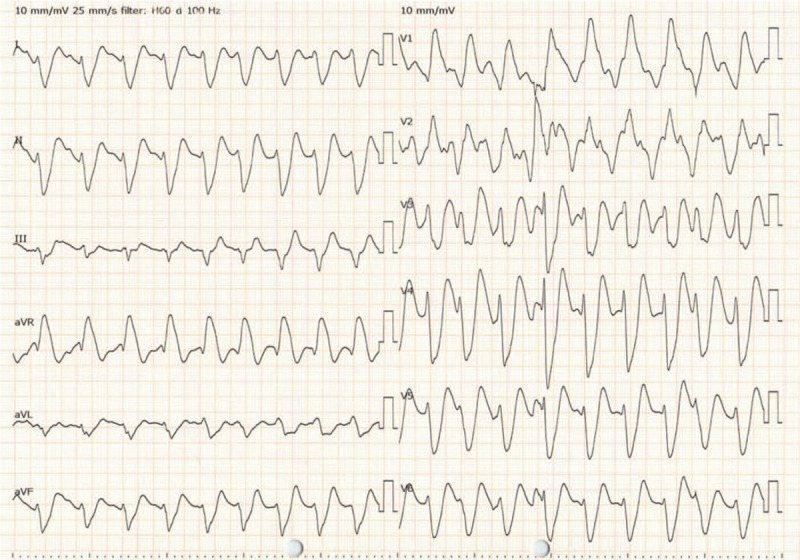
A 12-lead electrocardiogram of the case after intubation. It shows a wide QRS complex tachycardia and a prolonged QT interval (QRS complex duration, 234 ms; QTc (corrected QT) interval, 512 ms).

**Figure 2:**
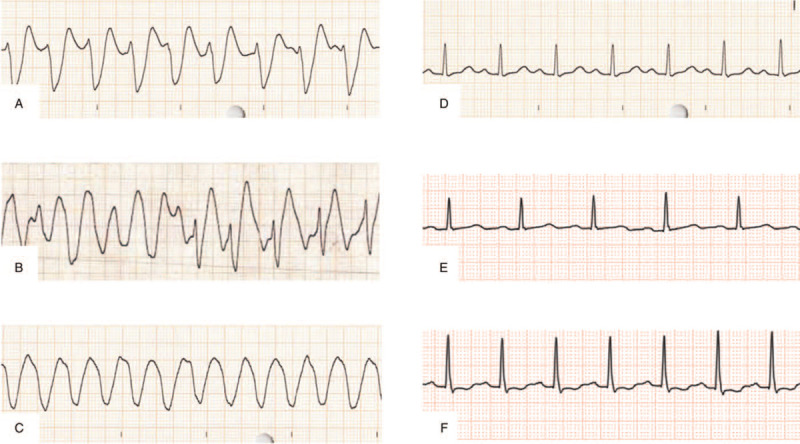
The electrocardiogram (lead II) time course of the case. (A) Recorded after intubation (the same as in Fig. 1). (B) Recorded at cardiac arrest: Indicates pulseless polymorphic ventricular tachycardia and pulseless electric activity. (C) Recorded shortly after starting the pump for VA-ECMO: Shows a ventricular tachycardia but no arterial pulse pressure. (D) Recorded 3 hours after starting the pump for VA-ECMO: Shows a normal QRS complex duration and a prolongation of the PQ and QT interval (QRS complex duration, 96 ms; PQ interval, 218 ms; QTc interval, 461 ms). (E) Recorded on day 2: Shows a normal PQ interval and a prolongation of the QT interval (PQ interval, 184 ms; QTc interval, 487 ms). (F) Recorded on day 4: Shows a normal QT interval (QTc interval, 429 ms).

His seizure stopped after intravenous administration of diazepam, and he was intubated because of his comatose status and generalized status epilepticus. After the intubation, he received an electrocardiogram (ECG), which displayed a wide QRS complex tachycardia and a prolonged QT interval (Figs. 1 and 2A: QRS complex duration, 234 ms; QTc (corrected QT) interval, 512 ms), and he suffered from cardiovascular instability. Despite synchronized cardioversion and an intravenous bolus of sodium bicarbonate (210 mEq), hemodynamically unstable refractory tachycardia persisted. Two hundred minutes after ingestion, his carotid artery was not palpated, and the ECG indicated polymorphic ventricular tachycardia (VT) and pulseless electric activity (Fig. 2B). Although he regained spontaneous circulation after 2 minutes of cardiopulmonary resuscitation, he had another cardiopulmonary arrest shortly afterward. The VA-ECMO procedure was initiated 240 minutes after ingestion based on the prediction that refractory arrhythmia and cardiac arrest could easily recur until the TCA-associated cardiotoxicity had disappeared. The VA-ECMO conditions were as follows: a 22 Fr drainage catheter passed from the right vein to the right atrium, and a 20 Fr arterial catheter was used for the left femoral artery. The pump was started at 2400 rpm, and the resulting blood flow was approximately 3.0 L/minute.

**Figure 3:**
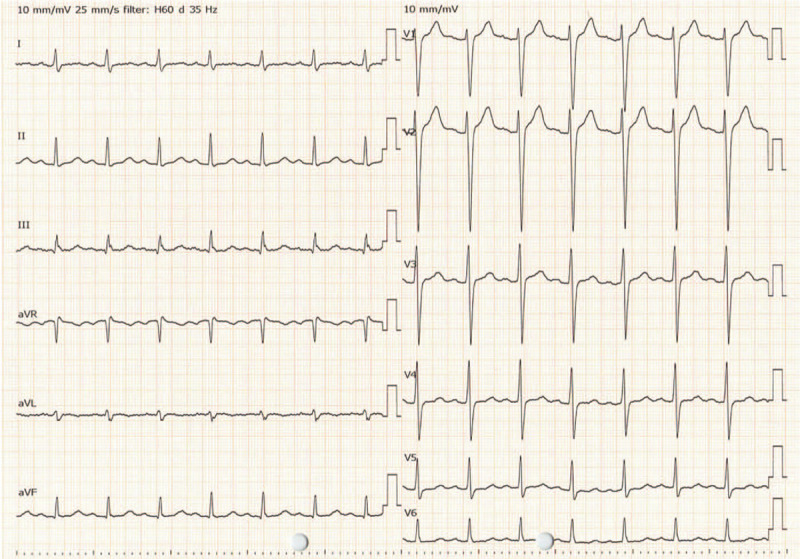
A 12-lead electrocardiogram of the case 3 hours after starting the pump for VA-ECMO. QRS complex duration was normal; the abnormal ratio of the R and S waves in the lead aVR had disappeared; the PQ and QT intervals were still prolonged (QRS complex duration, 96 ms; PQ interval, 218 ms; QTc interval, 461 ms).

After starting VA-ECMO, the patient's mean arterial pressure increased to 70 mm Hg without catecholamine use, but his arterial pulse pressure was not observed, and the ECG indicated a pulseless VT (Fig. 2C). His hemodynamic status stabilized on VA-ECMO. Three hours after initiating the pump for the VA-ECMO procedure, the patient regained his arterial pulse pressure (91/69 mm Hg without catecholamine), and the ECG returned to sinus rhythm with normalized QRS complex duration, although the prolongation of the PQ and QT interval remained (Figs. 2D and Fig. 3: QRS complex duration, 96 ms; PQ interval, 218 ms; QTc interval, 461 ms). The TTM was initiated with the VA-ECMO procedure using a target temperature of 34°C. Activated charcoal was administered via a nasogastric tube because it was predicted that he had ingested a large amitriptyline overdose that caused cardiotoxicity and even cardiac arrest.

**Figure 4:**
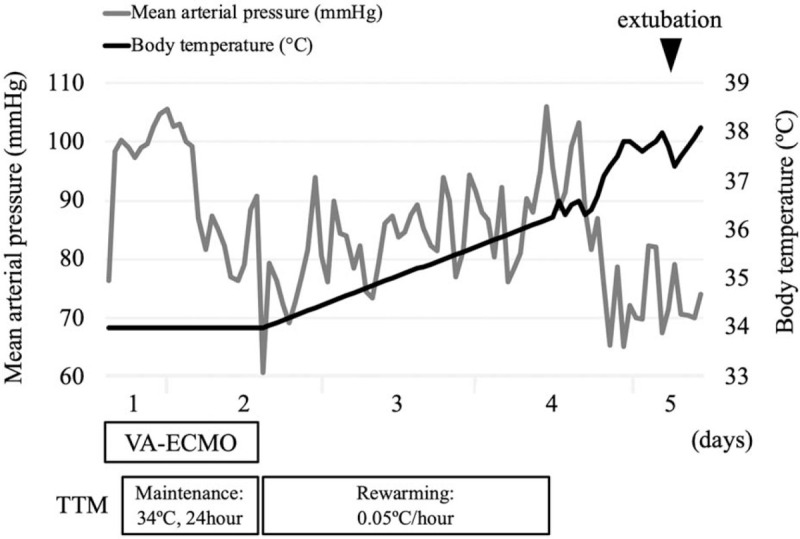
The clinical course of the case. The hemodynamic status stabilized after initiating the VA-ECMO pump, and ECMO was weaned off 27 hours after starting the pump. A target temperature of 34°C was maintained for 24 hours, followed by rewarming at a rate of 0.05°C/hour.

**Figure 5:**
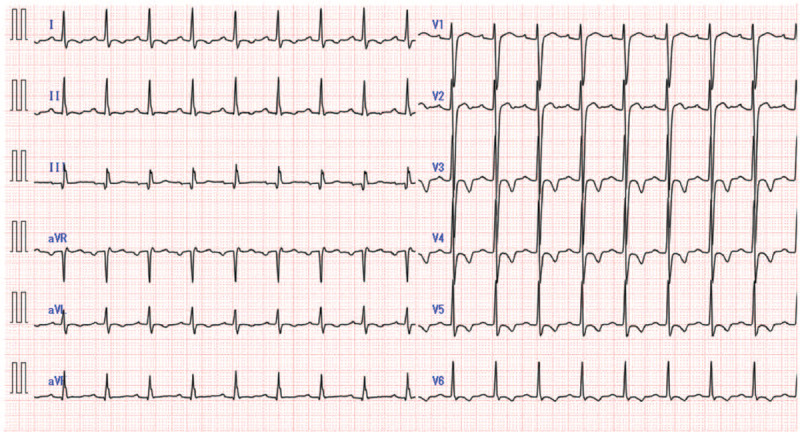
A 12-lead electrocardiogram of the case on day 4. QT interval was normalized (QTc interval 429 ms).

On day 2, the ECG did not show a recurrence of the ventricular arrhythmia and a prolongation of the PQ interval (Fig. 2E: PQ interval 184 ms, QTc interval 487 ms). Therefore, 27 hours after initiating the pump, the patient was weaned off the VA-ECMO. After the patient was weaned off the VA-ECMO, the temperature control method was changed to a surface cooling device. The targeted temperature was maintained at 34°C for 24 hours (including the temperature control period with VA-ECMO), followed by a gradual rewarming at 0.05°C/hour (Fig. 4). His hemodynamic status was stable after VA-ECMO weaning off, and the ECG displayed a normal sinus rhythm without a prolonged QT interval on day 4 (Figs. 2F and Fig. 5: QTc interval 429 ms). The TTM was completed after confirming that his body temperature was rewarmed to 36°C.

His mental status improved, and he was extubated on day 5. His general condition was good, and he moved to the psychiatric ward on day 8. He was discharged on day 15 without neurological impairment, and the postdischarge course was uneventful.

## 3 Discussion and conclusion

In this study, we report a case of TCA-induced cardiac arrest that was successfully resuscitated using VA-ECMO in combination with TTM. Although VA-ECMO and TTM are widely used in critical settings, only a few reports have documented cases of TCA intoxication that required intensive care, including VA-ECMO or TTM. We searched PubMed (up to November 2020) using the following terms: (“extracorporeal circulation” OR “extracorporeal membrane oxygenation” OR “percutaneous cardiopulmonary support”) AND (“tricyclic antidepressant”). We found 6 published cases of TCA intoxication (except for cases of polyintoxication) that required VA-ECMO support for cardiotoxicity, including the present case.^[5–8]^ The mean age of these 6 cases was 22.7 years (range, 1–37 years). To our knowledge, this is the first reported case of TCA-induced cardiac arrest that was successfully resuscitated using VA-ECMO combined with TTM. Our case provides 3 important clinical insights as follows.

First, VA-ECMO is effective in patients with TCA-induced cardiac arrest. A previous report showed that intoxication accounts for an extremely small percentage of the causes of introducing VA-ECMO (50/5, 263 cases, 0.95%).^[9]^ However, several reports have shown that VA-ECMO is a relatively safe therapeutic option and may improve survival in critically ill poisoned patients experiencing cardiac arrest and severe shock.^[3,10]^ The most likely reason for this result is that the pathology of intoxication is generally reversible, and an improvement of the crisis situations is expected to occur by removing the causative substance. The first-line treatments for patients with hemodynamic instability or wide QRS complex due to TCA-induced cardiotoxicity include fluid resuscitation, sodium bicarbonate administration, vasopressor administration, and antiarrhythmic-agent administration.^[11,12]^ VA-ECMO is considered in patients refractory to those interventions.^[11–13]^ In all cases presented in Table 1, including this case, the serum concentration of ingested drugs exceeded the therapeutic range, suggesting that TCA-induced cardiotoxicity can easily occur. Moreover, all cases went into refractory shock and cardiac arrest despite adequate fluid resuscitation and sodium bicarbonate administration, and all cases were successfully weaned from VA-ECMO. If VA-ECMO is available, clinicians should consider its early initiation in patients refractory to conventional therapies.

**Table 1:** Case reports of TCA-induced cardiac arrest requiring VA-ECMO support^[5–8]^.

	Age/Sex	Ingested drug	Serum concentration of drug on arrival (ng/mL)	Time from arrival to ECMO start (hours)	Duration of ECMO support (hours)	TTM	CPC/OPC	Reference
1	1, F	Desipramine	> 3,000	10.5	60	Not Stated	1, 1	^[5]^
2	37, F	Imipramine	Not measured	3.5	7	Not Stated	5, 5	^[6]^
3	26, F	Nortriptyline	1,100	1.8	14	Not Stated	1, 1	^[7]^
4	32, F	Imipramine	Not measured	14	23	Not Stated	1, 1	^[7]^
5	21, M	Amitriptyline	306.2	1.6	Not Stated	33°C, but discontinued	4, 4	^[8]^
6	19, M	Amitriptyline	2,902	1.5	27	34°C for 24 hours	1, 1	This case

Second, routine ECG screening during VA-ECMO is useful to assess the timing for weaning off the VA-ECMO support, as well as the degree of cardiotoxicity. The following ECG findings suggest an increased risk of seizure and ventricular arrhythmia:

1. prolongation of the QRS complex (QRS complex duration >100 ms),
2. prolongation of QT interval (QTc interval > 430 ms), and
3. abnormal ratio of the R and S waves in lead aVR (R-to-S ratio >0.7).^[11]^

In our case, the ECG on admission met these 3 criteria (Figs. 1 and 2A: QRS complex duration, 234 ms; QTc interval, 512 ms; R-to-S ratio, 3.0), suggesting that the occurrence of cardiotoxicity led to ventricular arrhythmia and cardiac arrest despite intravenous bolus of sodium bicarbonate. However, the ECG obtained 3 hours after initiating the pump for VA-ECMO displayed only a prolongation of the QT interval, suggesting that the risk of seizure and ventricular arrhythmia had decreased. A prolongation of PQ was observed at that time, but it was normalized on day 2 before weaning off the VA-ECMO. Previous reports described that improving the prolongation of the QRS complex duration or the PQ/QT interval, as along with the stabilization of the hemodynamic status, are necessary for weaning off the VA-ECMO, although a clear criterion for weaning off has not been established.^[5–7]^ Our case is consistent with previous reports, and routine ECG screening during VA-ECMO support will be a useful tool for determining the timing to wean off the VA-ECMO.

Third, TTM is safe in comatose survivors of cardiac arrest due to severe TCA intoxication. Potentially, TTM itself can aggravate the effect of amitriptyline by two mechanisms:

1. It affects the cardiac conducting system, resulting in a prolongation of the QT interval and the QRS complex duration,^[14]^ and
2. it decreases the activity of CYP2D6 (cytochrome P450 2D6), which metabolizes amitriptyline and nortriptyline.^[15,16]^

However, 1 report found that TTM was safe in a patient with drug-induced cardiac arrest.^[17]^ In this case, the target temperature was set at 33°C for 24 hours after resuscitation from cardiac arrest induced by amitriptyline and venlafaxine (serotonin-norepinephrine reuptake inhibitor), and serious cardiac arrhythmia was not observed during therapeutic hypothermia. In the context of safety and efficacy, our case suggests that TTM represents a reasonable strategy for survivors of cardiac arrest caused by acute intoxication.

## Acknowledgments

We thank all colleagues in the Emergency and Critical Care Center, Mie University Hospital (Drs. F. Okuno, R. Esumi, Y. Senga, T. Yamaguchi, T. Shinkai, A. Ito, D. Niimi, G. Miyamura, T. Kaneko, Y. Omori, M. Fujioka, and T. Takeda) for their assistance. We would like to thank Editage (www.editage.jp) for English language editing.

## Author contributions

**Conceptualization:** Kaoru Ikejiri.

**Supervision:** Hiroshi Imai.

**Writing – original draft:** Kaoru Ikejiri.

**Writing – review & editing:** Yuichi Akama, Yohei Ieki, Eiji Kawamoto, Kei Suzuki, Kazuto Yokoyama, Ken Ishikura.
